# Exosomes and Melatonin: Where Their Destinies Intersect

**DOI:** 10.3389/fimmu.2021.692022

**Published:** 2021-06-11

**Authors:** Adriana Alonso Novais, Luiz Gustavo de Almeida Chuffa, Débora Aparecida Pires de Campos Zuccari, Russel J. Reiter

**Affiliations:** ^1^ Health Sciences Institute (ICS), Mato Grosso Federal University (UFMT), Sinop, Brazil; ^2^ Department of Structural and Functional Biology, Institute of Biosciences, São Paulo State University (UNESP), Botucatu, Brazil; ^3^ Department of Molecular Biology, Cancer Molecular Research Laboratory (LIMC), São José do Rio Preto Medicine School (FAMERP), São José do Rio Preto, Brazil; ^4^ Department of Cell Systems and Anatomy, University of Texas (UT) Health, San Antonio, TX, United States

**Keywords:** extracellular vesicles, melatonin, exosomes, therapeutic potential, combination, diseases

## Abstract

Cell-to-cell communication is a broad and complex process associated with regular stimuli to maintain healthy cell interactions. One of the agents capable of cellular communication is known as an exosome, a subset of extracellular vesicles (EVs) released by the cell membrane. The exosome contains a wide range of functional proteins, mRNAs and miRNAs, which have the potential to interact with healthy or diseased cells in the body. On the other hand, melatonin also acts as a cellular communicator, produced and released by the pineal gland in a circadian way and also, non-circadian melatonin is derived from the mitochondria of all normal cells. In addition to exhibiting antioxidant, anti-inflammatory, anti-tumor and anti-aging activities, melatonin has recently been studied by its influence on exosomes. This review summarizes the relationship between exosomes and melatonin in various pathological processes. There is robust evidence that their combination ameliorates inflammation, ischemia-reperfusion injury, hepatic metabolic disturbance, cancer immunosuppression status, degenerative processes like chronic kidney disease, vascular calcification, ageing, ischemic brain injury, neurodegenerative diseases, obesity, colitis, wound healing and even embryonic development. Association of exosomes and melatonin represent a promising therapeutic tool, capable of interfering with basic molecular processes, such as oxidative stress and the inflammatory cascade, which support many pathophysiological aspects of diseases.

## Introduction

Effective means of communication have played crucial roles in society and are relevant factors for the development of humanity. Likewise, communication takes place at the cellular and molecular level, making organic homeostasis possible.

There are several mechanisms of cellular communication systematically described as being cell signaling pathways. So, this review will focus on two entities that have attracted significant attention in recent years: exosomes and melatonin. This relationship is due to their numerous signaling activities and crosstalk in eukaryotic organisms, and certainly, a deeper understanding of their actions will bring valuable benefits for science.

Recent progress on melatonin and exosome research will be outlined and the interactions between these two signaling pathways will be explored, with the potential to interfere in disease-related inflammatory, ischemic, degenerative, and neoplastic processes. In view of the association of these biomolecules, we propose that this partnership will benefit possible protective molecular mechanisms against pathological processes. Finally, the potential combined therapeutic use of exosomes and melatonin, working together for a healthy homeostasis, will be considered.

## Exosomes: Biogenesis and Biological Function

The first scientific description of exosomes dates from the 80s, when Johnstone and colleagues (1) observed that sheep reticulocytes lost their transferrin receptors during the maturation process to adult red blood cells; this occurred due to the release of small vesicles into the extracellular medium. In addition to transferrin receptors, their study provided evidence for the loss of selective membrane proteins during *in vitro* maturation of reticulocytes.

Exosomes are a subtype of extracellular vesicles (EVs) but the nomenclature of the different extracellular vesicles (EVs) has generated some confusion over time. However, it has been generally accepted that EVs may be classified into three groups according to their size and biogenesis, i.e., exosomes (30–200 nm), microvesicles (100–1000 nm) and apoptotic bodies (> 1000 nm)  (2–5). The *International Society for Extracellular Vesicles* recently published a position statement and update of *Minimal information for studies of extracellular vesicles* (2018 MISEV guidelines) considering “extracellular vesicle” the preferred generic term and recommending that subtypes must be defined by physical and biochemical characteristics and/or conditions/sources (6). In the present review, the exosome terminology will be used with respect to the referenced publication.

Exosomes originate from the endocytic pathway that initiate when the cytoplasmic membrane undergoes an invagination to form an early secretory endosome. Then, intraluminal vesicles (ILVs) are formed inside large multivesicular bodies (MVBs) and late endosomal maturation occurs by acidification. The last step is the release of the ILVs as exosomes through fusion with the plasma membrane (5, 7).

Although initially exosomes were thought to be involved merely in waste disposal (8), in 2007 Valadi (9) showed the use of exosomes by some cells to transfer genetic material between adjacent or distant cells. The transferred materials include mRNAs to make proteins and microRNAs to regulate the expression of genes, secreted by cells during normal and pathological conditions (10, 11).

The exosomal content includes proteins, DNA, mRNA, microRNA, long ncRNA and circular RNA, which play important roles in the regulation of growth, metastasis and angiogenesis. Since these processes are involved in cancer development, it is reasonable that exosomes are used as prognostic markers and as a basis for tumor graduation in patients with neoplasia (12, 13). Also, since the exosomes carry biomacromolecules from the source cell, they can represent a molecular bioprint of the original cell (14).

## Melatonin: Biogenesis and General Biological Functions

Melatonin (N-acetyl-5-methoxytryptamine) was first described in 1958 when Lerner and his colleagues (15), while looking for a treatment for vitiligo disease, isolated the active substance from the bovine pineal gland extract which was capable of lightening the skin of amphibians and inhibiting the melanocyte-stimulating hormone. Very soon thereafter, investigators observed that melatonin influenced the brain, and thereby, the gonads and other components of the neuroendocrine systems (16–18). The pineal gland produces melatonin in a circadian way, which explains its chronobiotic influence on the body’s endocrine and non-endocrine rhythms, such as the sleep cycle:wakefulness and reproduction (18, 19).

Scientific reports have shown, through the finding of melatonin in α-proteobacteria and photosynthetic cyanobacteria, that melatonin molecule evolved in bacteria that were phagocytosed by early eukaryotes for nutritional purposes. Thereafter, the bacteria appear to have developed a symbiotic relationship with the eukaryotic host, the α-proteobacteria evolving into mitochondria while the cyanobacteria evolved into chloroplasts and these organelles continue to exist until today (20, 21).

The synthesis of melatonin is derived from the amino acid tryptophan, serotonin being an intermediate compound. Two main enzymes control melatonin synthesis: arylalkyl N-acetyltransferase (AANAT) and acetylserotonin O-methyltransferase (ASMT) (22, 23). Initially it was believed that the presence of melatonin in all cells and its main effects were related to their absorption from the blood. However, it was later discovered that many cells are probably capable of carrying out the enzymatic conversion of serotonin to melatonin (19, 24–26).

Although many functions of melatonin are mediated by membrane receptors, it is now known that there are functions independent of its receptors. Melatonin and its metabolites are transported to cells *via* the cell oligopeptide transporter (PEPT) 1/2 ‘, the organic anion transporter (OAT) 3 and the glucose transporter (27, 28). It is believed that transport *via* PEPT1/2 may be involved with its oncostatic effects (27). Melatonin is also transported into the mitochondria by means of PEPT1/2, although it is also synthesized by probably all cellular mitochondria (29–31).

The first report on the potent action of melatonin as a scavenger of direct free radicals occurred 30 years ago (32). Its efficiency as a powerful antioxidant stems from its ability to stimulate several antioxidant enzymes, in addition to directly neutralizing a series of free radicals and reactive oxygen and nitrogen species (33). Melatonin interacts with the highly toxic hydroxyl radical (· OH) at a constant rate equivalent to that of other highly efficient hydroxyl radical scavengers (32). Additionally, there are assumptions that melatonin neutralizes hydrogen peroxide, singlet oxygen, peroxynitrite anion, nitric oxide and hypochlorous acid (34). Superoxide desmutase, glutathione peroxidase, glutathione reductase and other antioxidant enzymes are also stimulated by melatonin (29, 35–39).

Melatonin has recently been reclassified as a multitasking molecule, and not exclusively a hormone, due to the finding about the existence of essential enzymes for its synthesis and the presence of melatonin receptors in many tissues and, also, the discovery of its antioxidant and generalized anti-inflammatory properties (40, 41).

Melatonin functions as a glycolytic molecule which inhibits pathological aerobic glycolysis of diseased cells allowing them to resume normal mitochondrial oxidative phosphorylation; this change converts pathological cells to a healthier phenotype. It is likely that the glycolytic function of melatonin explains its protective actions against a variety of diseases (33).

## Exosomes and Melatonin: Compatible Partners

**Figure 1:**
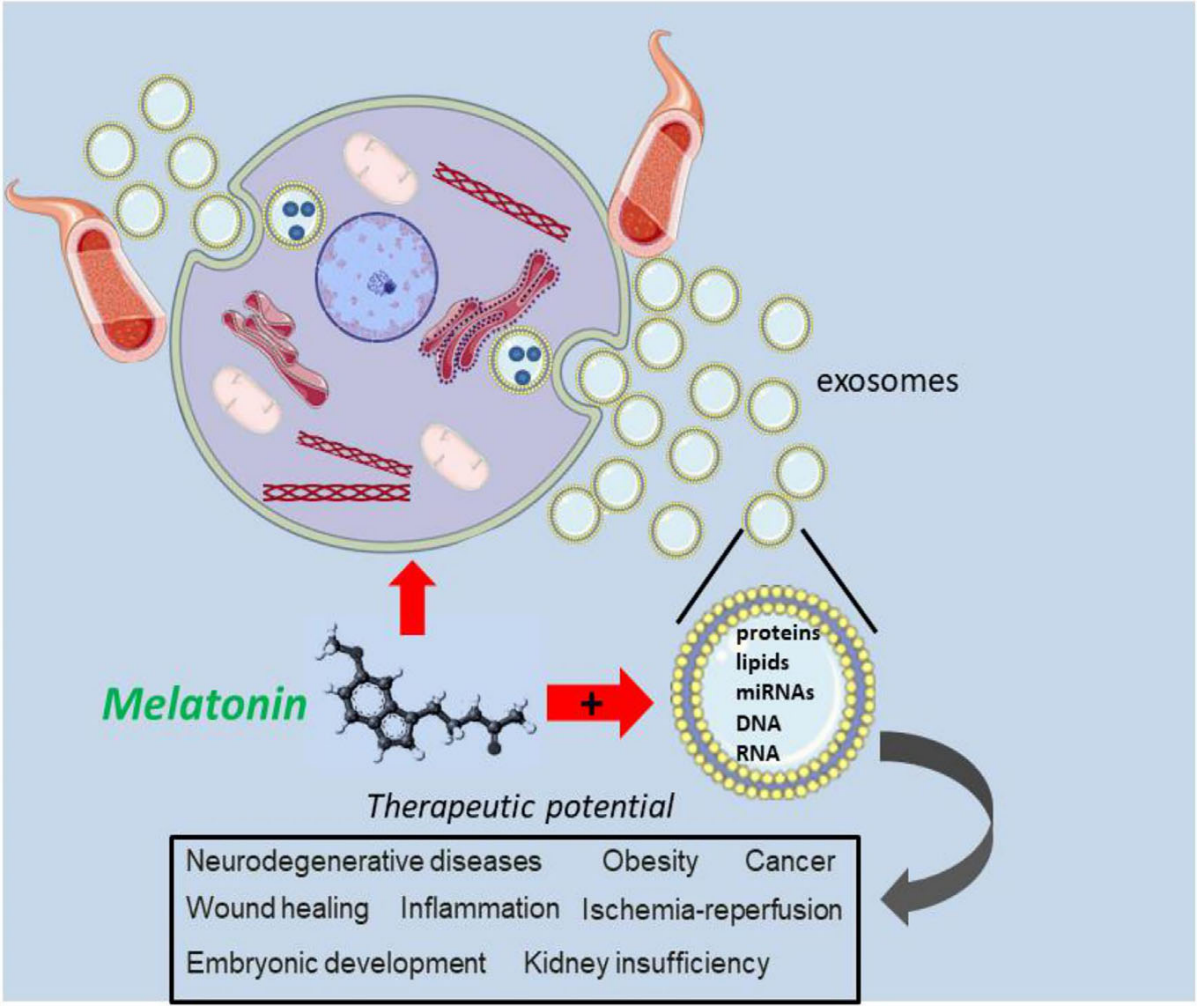
The combination of melatonin and exosomes is promising therapeutic strategy. By acting directly on damage cells, melatonin is thought to intracellulary interface with exosome trafficking, possibly by modifying their molecular contrent. The user of melatonin in combination with an exosomes may be an important approach to alter the biology of exosomes related to dysregulated signaling pathways in several pathologies and disease conditions.

Since exosomes express many different surface receptors, their influence on recipient cells differs functionally; this results in sets of exosomes that are capable to induce cell survival, others that influence the apoptosis process and still others that interfere with immunomodulation, in different types of target cells (42). The exosomes achieve these effects mediating an autocrine and paracrine intercellular cross-talk that, subsequently, promotes a modification of both local and distant microenvironments (43). Besides common core proteins that are a reflection of their biogenesis, many different proteins carried by the exosomes reflect the original cells related to the phenotypic and physiological state, meaning that they may give important information on the pathological processes of many medical entities (44). In turn, melatonin combined with exosomes showed beneficial effects, like suppressing oxidative stress and apoptosis *in vitro* and inflammation, oxidative stress, DNA/mitochondrial damage, and apoptosis *in vivo* (45). Also, this therapeutic combination has exhibited an important protection of nervous system through the regulation of the TLR4/NF-κB signaling pathway (46). Also, exosome-melatonin therapy was found to mitigate vascular calcification and aging (47) and other beneficial therapeutic actions, that will be the scope of this review. For example, Xia and colleagues (48) demonstrated that melatonin may induce the polarization of macrophages by changing exosomal contents released from adipocytes. **Figure 1** summarizes the therapeutic potential of melatonin in various pathological conditions.

The mitochondria play a central role in almost every activity a cell undertakes; it is the preferential site of ATP synthesis and reactive oxygen species (ROS) generation. Mitochondria can take up pineal-derived or exogenously administered melatonin from the circulation, but they also have the ability to intrinsically produce it (49–51). Mitochondria are believed to have evolved when melatonin-producing bacteria were engulfed by early eukaryotes (21, 26). When confronted with melatonin from the blood, the mitochondria concentrate it against a gradient (52).

**Figure 2:**
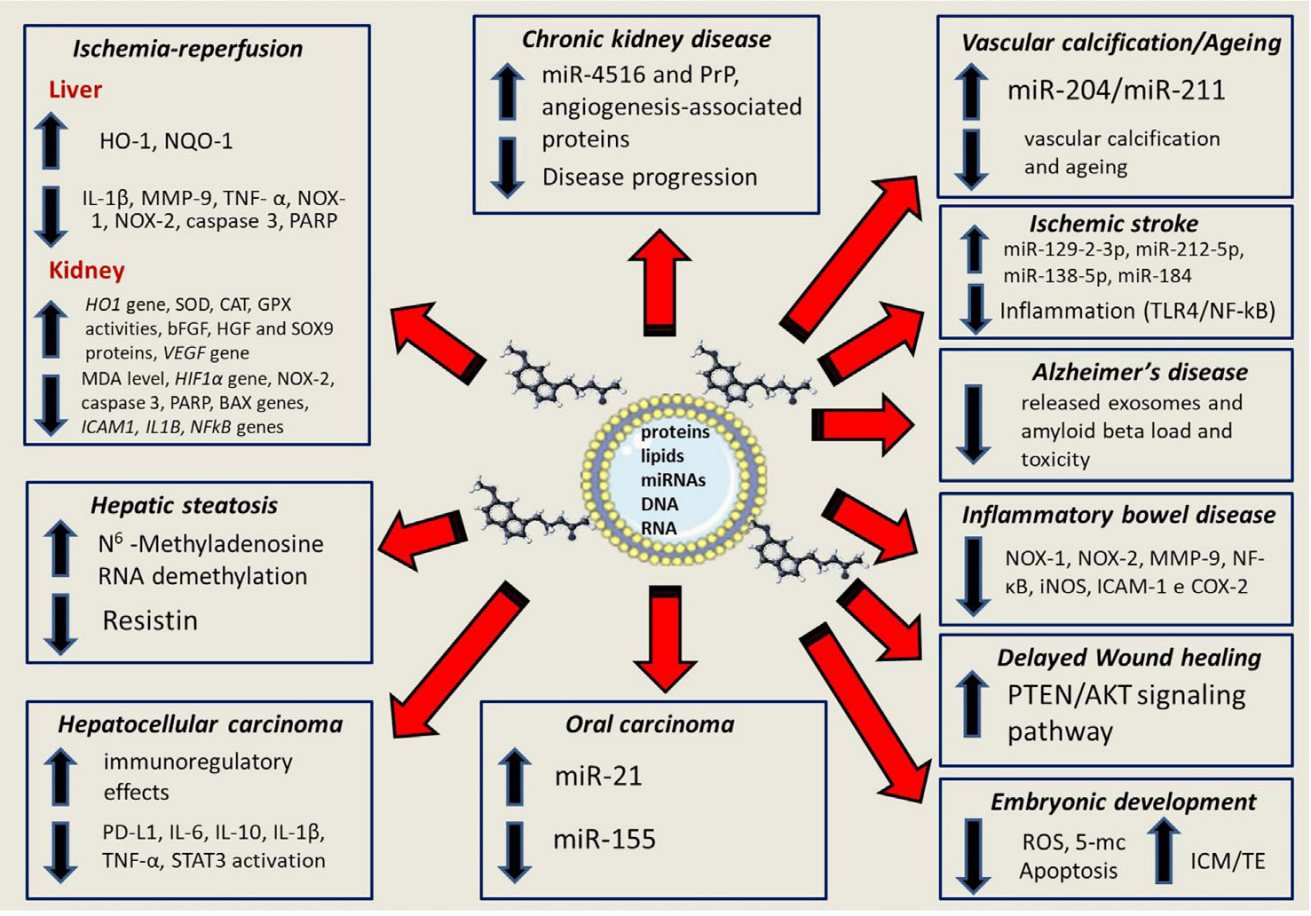
The up- and down-regualtion of multiple process that involve the partnership between melatonin and exosomes; these include signaling pathways for inflammation, oxidative stress and apoptotic events as well as well as gene. microRNAs, protein and transcription factors. An in-depth knowledge of the control of the synergistic effects between melatonin and exosomes offers a valuable therapeutic promise for the treatment of several diseases.

It is believed that the transfer of information between cells can be mediated by the mitochondrial genome transfer and even the entire mitochondria (53). The research of Guescini and colleagues (54) provided evidence that glioblastoma and astrocyte cells release exosomes carrying mitochondrial DNA (mtDNA), which can be transferred to other cells. The role and relevance of exosomes in mitochondrial homeostasis is an emerging area of research, which may clarify many details of this interrelation (53, 55). **Figure 2** summarizes some of the many molecular processes regulated by melatonin and exosomes in various pathological conditions.

## Exosome-Melatonin Therapy Protects Against Ischemia-Reperfusion Injury

Hepatic ischemia-reperfusion (I/R) injury is a major complication of liver tissue often observed during liver surgery (e.g., liver resection, trauma, and liver transplantation) with pro-inflammatory components and immune cells being important players. For patients undergoing surgeries that result in prolonged ischemic intervals or liver graft transplantation, a deeper understanding of liver I/R injury will reflect improvements in the clinical care of patients (55). Recently, Sun et al. (45) examined liver ischemia/reperfusion (LIR) injury and confirmed that combined melatonin and exogenic adipose mesenchymal stem cell (ADMSC)-derived exosome treatment offered superior protection against I/R injury, when compared to the isolated treatment. In vitro studies used a macrophage cell line (RAW), pre-treated with lipopolysaccharides, and hepatocytes previously incubated with melatonin or exosomes before the hypoxia lesion. These studies showed that exosomes alone and in combination with melatonin, respectively, cause suppression of inflammation by reducing MIF, MMP-9, IL-1β, TNF-α, COX-2 and oxidative stress markers (NOX-1, NOX-2, oxidized protein), in addition to apoptotic molecules (cleaved caspase 3 and PARP). In addition, *in vivo* experiments analyzed liver specimens from male adult Sprague-Dawley rats (n = 50) equally categorized into the following experimental: (1) sham controls (SC), (2) LIR only, (3) LIR-exosome (intravenous administration of 100 µg, 30-minute post-LIR), (4) LIR-melatonin (intra-peritoneal administration of 20 mg/kg, 30 minute post-LIR and 50 mg/kg at 6 and 18 hours post-LIR), and (5) LIR-exosome-melatonin groups. The authors demonstrated that I/R animals treated with the combination of exosomes and melatonin had lower plasma AST concentrations and liver injury scores, when compared to other groups. Inflammatory markers (ICAM-1, IL-1β, MMP-9, TNF-α, NF-κB, RANTES), cellular immunoregulatory molecules (CD3 +, CD4 +, CD8 +, CD161 +, CD11 +, CD14 +, F4/80), expression of molecules related to apoptosis (cleaved caspase-3, PARP), oxidative stress (NOX-1, NOX-2), DNA damage (γ-H2AX) and markers of mitochondrial damage (cytosolic cytochrome- C) exhibited a similar pattern to those of liver injury scores. On the other hand, after the combined treatment, the expression of antioxidant proteins (HO-1, NQO-1) exhibited progressive increases in stem cells. The most relevant findings were that the plasma AST level and liver injury score were significantly suppressed in animals that suffered I/R injury after exosome or melatonin therapy, and still increased significantly after the combined treatment with exosomes and melatonin.

On the other hand, renal ischemia-reperfusion injury (RIRI) and its dysfunction syndrome has the potential to accelerate the development of chronic kidney disease (CKD). The release of free radicals, mitochondrial dysfunction, induction of apoptosis and inflammation are among the main causes of RIRI (56). Hence, therapeutic agents targeting oxidative stress, apoptosis, and inflammation may be beneficial in clinical approach to RIRI. In his research, Alzahrani (57) evaluated the effectiveness of exosomes derived from mesenchymal stem cells (MSCs) preconditioned with melatonin. He noted that the combined treatment provided the best protection against I/R injury when compared to therapy with MSCs or exosomes derived from MSCs that had not been preconditioned. There was a significant improvement in I/R after all treatments (MSCs, Exo and Exo + Mel), but the improvement in the Exo + Mel group was evidently greater. To assess the degree of protection afforded by the therapy, they took into account some issues such as histopathological score, blood levels of urea nitrogen in the blood (BUN) and creatinine, the state of oxidative stress (MDA level, HIF1α gene and NOX2 protein), the activities of antioxidant enzymes (HO1 gene and SOD, CAT, GPX activities), evidence of apoptosis (caspase 3 activity and PARP1, BAX genes), anti-apoptotic effect (BCL-2 gene), degree of inflammation by reduction of MPO activity and low expression of the ICAM1, IL1B, NFkB genes), intensity of regeneration (bFGF, HGF and SOX9 proteins) and, finally, the degree of angiogenesis (VEGF gene). This exosome ameliorative effect was mediated, at least in part, by inhibition of the oxidative stress, apoptosis, and inflammation, while inducing antioxidant properties, regeneration and angiogenesis, resulting in improved renal repair and function.

The therapeutic value of melatonin (20 mg/kg body weight/day) given for three days, *via* intraperitoneal injection, in association with administration of mesenchymal stem cells, and their extracellular vesicles, have also been tested in RIRI (58). Initially, the authors established the renal I/R model and subsequently injected mesenchymal stem cells or exosomes into both renal arteries during reperfusion. They observed an improvement in RIRI compared to different treatments, using parameters such as histopathological score, serum levels of urea, creatinine and retinol-binding protein, lipid peroxidation marker malondialdehyde, superoxide dismutase and catalase activities, and degree of apoptosis (less damage to DNA and protein X associated with B 2 cell lymphoma and higher B 2 cell lymphoma (genes/proteins). The researchers reported a remarkable inhibition of inflammatory markers and kidney damage (e.g., tumor necrosis factor alpha, interleukin-1β, kappa nuclear factor B, kidney injury molecule-1, IL-18, matrix metalloproteinase 9 and lipocalin neutrophils associated with gelatinase). The combined treatment of melatonin + exosomes was more effective, that is, lower scores for kidney damage, oxidative stress, inflammation and renal apoptosis parameters. The authors concluded that the combination therapy with melatonin, mesenchymal stem cells and exosomes has potential use in order to minimize the damage of renal I/R in rats.

## Exosome-Melatonin Therapy Protects Against Hepatic Metabolic Disturbance

Fatty liver is caused by the impregnation of more than 5% of the liver with fat. Although the moderate accumulation of triacylglycerol in the liver can exert a hepatoprotective effect, when this storage of hepatic lipids becomes prolonged, it can cause metabolic dysfunction, inflammation and advanced forms of non-alcoholic fatty liver disease (NAFLD) (59). Metabolic stress acquired due to disorders such as abnormalities in glucose and lipid metabolism, insulin resistance and inflammation can cause the development of NAFLD. NAFLD is usually associated with metabolic comorbidities, such as obesity, diabetes mellitus and dyslipidemia (60).

The role of melatonin in reversing hepatic steatosis was studied by Rong et al. (61), who suggested that crosstalk originating from adipose tissue could be a valid regulatory route. Melatonin supplementation caused a significant reduction in the amount of exosomal resistin derived from adipocytes. They later demonstrated that resistin was a fundamental cytokine to suppress phosphorylation of protein kinase α activated by 5 ‘adenosine monophosphate, capable of triggering stress in the endoplasmic reticulum, which resulted in hepatic steatosis. Melatonin reduced the production of resistin in adipocytes, through the brain and inhibition of protein 1 transcription similar to muscle arnt. Melatonin was able to improve the demethylation of the N6-methyladenosine RNA to degrade the resistin mRNA in adipocytes. In general, melatonin caused a reduction in the traffic of the exosomal resistin generated by adipocytes to hepatocytes, causing greater relief of stress-induced liver steatosis in the ER. Recent scientific indications, based on the study of new regulatory pathways mediated by melatonin, indicate that adipocyte-derived exosomes are a potential new target for the treatment of obesity.

## Exosome-Melatonin Therapy Changes the Neoplastic Immunosuppression Status in Hepatocellular and Oral Squamous Cell Carcinomas

Hepatocellular carcinoma (HCC) is the most common malignant tumor of the liver and has been linked to a high rate of death in cancer patients worldwide. Liver cirrhosis is considered the most important risk factor for the development of HCC, caused by chronic inflammation resulting from continuous hepatocyte damage, such as that which occurs in cases of hepatitis B and C (62). In HCC cells, exosomes favor microenvironment communication, and provide a fertile environment for cell proliferation and metastasis; how miRNAs, lncRNA, and proteins are exosome-sorted in a specific cell type remains unclear (63).

To assess the effects of exosomes derived from hepatocellular carcinoma (Exo-con) and exosomes derived from HCC cells treated with 0.1 mM melatonin (Exo-MT) on the expression of inflammatory factors and programmed death ligand 1 (PD-L1), Cheng et al. (64) devised an experiment using co-culture of Exo-con and Exo-MT with differentiated macrophages from THP-1 cells or RAW264.7 cells. The researchers observed that in macrophages co-cultured with Exo-MT there was suppression of the expression of PD-L1, while in those co-cultured with untreated exosomes (Exo-con) there was an increase in the expression of PD-L1 and levels cytokines, such as IL-6, IL-10, IL-1β and TNF-α. Therefore, the authors concluded that exosomes treated with melatonin were able to efficiently attenuate the expression of PD-L1 and the secretion of cytokines, by decreasing the activation of STAT3, promoting the immunoregulatory effects in HCC.

The oral cavity is the anatomical location where approximately 50% of the head and neck cancers (HNC) occur. The oral squamous cell carcinoma (OSCC) and epidermoid oral carcinoma are the most frequent malignant cancers, representing 90–95% of cases. The challenging paradigm is due to the worse prognosis and high mortality rate, combined with the lack of better perspectives (65). Differential microRNA (miRNA) expression profile in OSCC-related EVs have been documented both *in vitro* and in clinical experiments (66, 67). Hunsaker et al. (68) tried to identify the role of melatonin in oral carcinoma-associated EVs (including the exosomes) and impact on microRNA (miRNA) content in various oral cancer cell lines. They observed that melatonin significantly suppressed the expression of miR-155 in all OSCC-related extracellular vesicles. Moreover, the expression of miR-21 was significantly increased and no significant changes in miR-133a expression were observed, after melatonin administration in the three isolated OSCC. These results suggested a differential modulation of specific miRNAs, such as miR-21, miR-133a, and miR-155. In other words, melatonin differentially modulates miR-expression in OSCC extracellular vesicles, including exosomes.

## Exosome-Melatonin Therapy Is Helpful for Patients With Chronic Kidney Disease

Chronic kidney disease (CKD) is a clinical syndrome that occurs as a consequence of kidney function and/or structure deterioration. Unfortunately, it is an irreversible process with a slow and progressive evolution (69). One of the most important contributors in the pathophysiology of CKD and hypertension is the intrarenal renin-angiotensin system (RAS), due to the processes of inflammation and fibrosis. Studies using animal models of CKD have shown that the activation of intrarenal RAS and the consequent kidney injury can be mitigated by exogenous administration of melatonin, due to its antioxidant effects (70). Other studies have reported the use of isolated EVs from MSCs to prevent the progression of CDK in animal models and in patients (71, 72). Recently, Yoon et al. (73) isolated exosomes derived from healthy melatonin-treated MSCs. Comparatively, they evaluated the biological functions of exosomes derived from MSCs of patients with CKD, also treated with melatonin. They found that the treatment with melatonin increased the expression of cellular prion protein (PrP) in exosomes isolated from MSCs through the positive regulation of miR-4516. In addition, exosomes from melatonin-treated MSCs were able to protect mitochondrial function, cell senescence and the proliferative potential of MSCs, significantly increasing the level of proteins associated with angiogenesis in MSCs. With this study, the authors suggested a regenerative potential in the use of MSCs treated with exosomes and melatonin in patients with CDK through the miR-4516-PrP signaling axis.

## Exosome-Melatonin Therapy Attenuates Vascular Calcification, Ageing, Ischemic Brain Injury, and Neurodegenerative Diseases

Vascular calcification occurs in the elderly with comorbidities such as atherosclerosis, hypertension, diabetes, macroangiopathy and chronic kidney disease. The degree of vascular calcification is directly related to cardiovascular mortality and tissue amputation, being an important factor in determining the health of the elderly (74).

The role of exosomes in angiogenesis, vascular calcification and senescence of vascular smooth muscle cells (VSMCs) has been considered important in some studies (75). A study by Xu et al. (47) demonstrated that melatonin (10 μM) can attenuate both vascular osteogenic differentiation and VSMC senescence. In addition, they demonstrated that exosomes isolated from VSMCs or calcified vascular smooth muscle cells (CVSMCs), treated with melatonin, can be absorbed by VSMCs and attenuate osteogenic differentiation and senescence of VSMCs or CVSMCs, respectively. In addition, they demonstrated the mediation of the paracrine effects of exosomes secreted by VSMCs by exosomal miR-204/miR-211, through the BMP2 gene. The authors also found that treatment with melatonin relieved vascular calcification and aging in mice submitted to 5/6 nephrectomy plus a high phosphate diet (NTP 5/6). They were able to detect by fluorescence images that exosomes derived from VSMCs treated with melatonin were internalized in the arteries of mice, promoting a reduction in vascular calcification and aging. They reported that these effects were largely abolished by inhibition of exosomal miR-204 or miR-211. Thus, the authors concluded that exosomes obtained from VSMCs treated with melatonin were able to attenuate vascular calcification and paracrine aging through an exosomal miR-204/miR-211 dependent mechanism.

Ischemic stroke or cerebral ischemia is caused by the interruption of blood flow due to the blockage in an artery that supplies blood to the brain. Then, the reduction in cerebral oxygenation leads to permanent neural damage or death of neuronal cells if the circulation cannot be quickly restored (76). There is a need to find new neuroprotective substances, because although most pharmacological neuroprotectors have been effective in experimental studies, they have failed in clinical trials. Furthermore, it is important that these new substances can act on brain repair, supporting the concept of brain plasticity (77).

In this context, Wang et al. (46) decided to investigate the therapeutic potential of plasma exosomes and melatonin in the damage to the nervous system caused by I/R. They treated rats with melatonin and isolated plasma exosomes using a model of focal cerebral ischemia. They reported that inflammatory responses induced by ischemia and inflammasome-mediated pyroptosis were influenced (suppressed) by the treatment with plasma exosomes. In other words, melatonin increased the therapeutic effects of plasma exosomes, with a reduction in the intensity of the infarction and an improvement in recovery through the regulation of the TLR4/NF-κB signaling pathway. The authors also linked the altered miRNA profile in plasma exosomes treated with melatonin with the regulatory mechanisms involved in neurological recovery after ischemic injury.

Also, organic aging is directly related to oxidative stress, which is a condition that involves an imbalance between the formation of reactive oxygen species (ROS) and the cellular antioxidant capacity. With aging, ROS generation increases, but the organic antioxidant system gradually becomes dysfunctional. Thus, aging and various pathological conditions and human diseases, especially neurodegenerative diseases, are caused by biochemical changes in these macromolecular components, ultimately causing oxidative damage (78).

One of the most important neurodegenerative diseases is Alzheimer’s disease (AD), characterized by severe neuronal loss in the brain, leading to cortical dementia with an intense memory deficit (79). The pathophysiology of synaptic loss and neurodegeneration involves problems in the cleavage of the amyloid precursor protein (APP) with the production of APP beta-amyloid (Aβ) fragment, together with the aggregation of the hyperphosphorylated tau protein. In these individuals, the existence of comorbidities, metabolic, vascular and inflammatory changes, are essential factors for the disease process (80). In addition, the inducing role of exosomes in the spread of neurodegenerative diseases such as AD, due to the spread of toxic proteins has been studied (81–84). The studies have shown that the spread of neuropathology appears to be mediated by exosomes containing different forms of tau, especially the phosphorylated form of the tau protein carried by the exosomes (85, 86). However, as the influence of melatonin on the quantity and content of exosomes released from cells was still unknown, Ozansoy et al. (87) developed an *in vitro* Aβ toxicity model to investigate the possible role of melatonin treatment in exosome release and exosomal tau content. The experiment demonstrated that treatment with melatonin suppressed the number of exosomes released, and consequently the amyloid beta load and toxicity, acting by blocking the secretory molecules of EVs. In addition, they demonstrated that the timing of melatonin administration, whether before or after the application of Aβ, also affected the level of tau carried by the exosomes. The authors concluded that the study provided a starting point for the development of AD treatment strategies through the influence of melatonin on the exosomes.

## Exosome-Melatonin Therapy May Control the Inflammatory Process Associated With Colitis and Obesity and Assist in Wound Healing

Ulcerative colitis (UC) and Crohn’s disease are chronic inflammatory bowel diseases (IBD), with unknown etiology and partially understood pathogenesis. The process begins in the rectum and progresses involving different extensions of the colon mucosa. Typically, clinical signs such as rectal bleeding and diarrhea are observed. The potential use of exosomes in IBD diagnostic and treatment strategies is based on the knowledge that they influence the main pathways related to IBD, such as immune responses, barrier functions and intestinal flora (29). Thus, Chang et al. (88) proposed to elucidate the relationship between melatonin and exosomes in acute inflammatory colitis (CIA). In a model of AIC induced by sodium dextran sulfate (DSS) in rats, they tested the hypothesis that AIC could be suppressed by combining melatonin and exosomes released by mesenchymal stem cells derived from adipose tissue. They treated Sprague Dawley rats with doses of 50 mg/kg of melatonin on day 5 and 50 μM/kg of exosomes on days 5, 7 and 10, and also with the combination of melatonin with exosomes. They observed that the number of circulating inflammatory cells was lower in animals treated with melatonin-exosomes, when compared to those treated with melatonin or exosomes alone. The combination of melatonin and exosomes mitigated the effects of AIC induced by DSS, evidenced by a reduction in the expression of inflammation markers, oxidative stress, apoptosis and fibrosis. The combined treatment was also able to reduce colon injury scores, expression of inflammatory markers and DNA damage.

Obesity is a complex chronic disease with inflammation as a central and reversible process. The adipose tissue undergoes infiltration and activation of immune cells, particularly macrophages, capable of communicating with adipocytes and bringing these changes to the adipose tissue microenvironment (89, 90). In order to modulate the process of inflammation in obesity, Liu et al. (91) studied the effects of melatonin at a dose of 20 mg/kg/day for 14 days and reported that melatonin relieved inflammation while raising levels of α-ketoglutarate (αKG) in adipose tissue of obese mice. They revealed that αKG was the target for the inhibition of melatonin-mediated adipose inflammation, by promoting mitochondrial isocitrate dehydrogenase mRNA 2 (Idh2) elevation in adipocytes, resulting in an increased level of αKG. In addition, the researchers observed that sirtuin 1 (Sirt1) interacted physically with HDI2, forming a complex to increase the circadian amplitude of Idh2 and the content of αKG. Melatonin was able to effectively promote the secretion of exosomes from adipocytes, increasing the level of exosomal αKG derived from adipose tissue. They also reported an increase in the proportion of M2 to M1 macrophages, under the action of melatonin, transporting exosomal αKG to macrophages and promoting TET-mediated DNA demethylation. In addition, exosomal αKG attenuated signal transducers and transduction-3 (STAT3)/NF-κB activators through its receptor 1 oxoglutarate (OXGR1) in adipocytes. In conclusion, the authors suggested that melatonin had the potential to relieve metabolic inflammation because it may promote an increase in αKG cellular and exosomal levels in adipose tissue.

The healing difficulties caused by diabetes have an important influence on surgical results and may eventually develop into chronic wounds. Recent research has shown that the polarization of macrophages plays an important role in the healing process of diabetic wounds (92). To investigate the influence of melatonin and exosomes on the polarization of macrophages, Liu et al. (93) studied whether exosomes derived from mesenchymal stem cells (MSCs), pretreated with melatonin (MT) (Exo-MT), could have beneficial effects on the healing of diabetic wounds, when compared to untreated exosomes. The researchers observed that MT-Exo improved the healing of diabetic wounds by suppressing the inflammatory response, which was achieved due to the increase in the M2 to M1 polarization ratio, by activating the PTEN/AKT signaling pathway.

Also, in order to assess the potential effects of exosomes released by adipose tissue MSCs on inflammatory modulation, Heo et al. (94) examined the changes in anti-inflammatory genes, along with the polarization of M2 macrophages in fibroblasts treated with pro-inflammatory cytokines and THP-1 cells. They observed that exosome treatment positively regulated the expression of anti-inflammatory mRNA associated with M2 macrophages, in an inflammatory environment treated with gamma interferon and tumor necrosis factor alpha. The increase in anti-inflammatory modulation exerted by exosomes treated with melatonin occurred through exosomal miRNAs (for example, miR-34a, miR-124 and miR-135b).

## Exosome-Melatonin Therapy Benefits Embryonic Development

Embryo quality and even offspring development require ideal growing conditions. It is known that these conditions are influenced by oviductal EVs, which interfere in the interactions between the oviduct and the embryos. The absence of EVs in cell cultures is associated with inferior embryonic development. Coincidentally, it has been reported that melatonin is also abundantly present in oviduct fluids and EVs derived from oviduct fluid (95). It is also known that the addition of EVs or melatonin, alone, led to a significant negative regulation of ROS and 5-methylcytosine (5-mC), as well as an increase in the proportion of embryos in the blastocyst stage. The combined treatment with EVs and melatonin led to the same results, but there was a significant decrease in the apoptosis index and an increase in the internal cell mass index (ICM)/tropectoderm (TE). In conclusion, the research provided insights into the role of EVs and melatonin in cellular communication and between embryos and the oviduct.

## Concluding Remarks and Perspectives

Delaying disease processes or stage advances by simply activating or correcting specific cellular mechanisms of disease would be a monumental development. Although still *via* unknown mechanisms, science is opening up the field of regenerative medicine, in which quiescent elements present during illness can themselves be activated to reverse the processes of disease.

Regenerative medicine is attractive to the medical community since many scientific reports provide evidence that human body is specially equipped to heal itself. Within the context of organic elements capable of promoting self-healing are melatonin and exosomes, which can significantly interfere in the main processes involved with cell ageing and molecular damage.

We explored herein the promising results of the therapeutic use of exogenous melatonin combined with exosomes derived from several tissues (e.g., mesenchymal stem cells), and in many pathological conditions such as ischemia-reperfusion injury, obesity, hepatocellular carcinoma, oral squamous cell carcinoma, chronic kidney disease, ischemic brain syndrome, neurodegenerative diseases like Alzheimer’s disease, acute inflammatory colitis, diabetes-related wound healing, vascular calcification, ageing, and also in embryonic development. The evidence suggests that by changing the exosomal content and load, melatonin may potentially improve the nature of exosomes associated with diseases with recognized molecular background.

Based on the above-mentioned studies, we reiterate that melatonin-exosome combinations reveal important and novel therapeutic features possibly affecting miRNAs, proteins, and lipids related to degenerative, inflammatory, and neoplastic disorders. Moreover, melatonin-exosome therapy could fight most of these disease processes by preventing their progression, not only their clinical manifestations. Finally, melatonin/exosome-based therapy presents numerous advantages over traditional treatments, since melatonin has no adverse effects, is well-tolerable, and possesses powerful anti-oxidant, anti-inflammatory, and anti-ageing properties.

## Author Contributions

AN compiled the literature and wrote the manuscript. LC prepared figures for the article, guided the insertions and the scope of the review. RR and DZ conceived the review and checked all drafts and the final version of the report. All authors contributed to the article and approved the submitted version.

## Conflict of Interest

The authors declare that the research was conducted in the absence of any commercial or financial relationships that could be construed as a potential conflict of interest.
